# Emotional intelligence and spirituality in university students: an integrative review

**DOI:** 10.3389/fpubh.2026.1819327

**Published:** 2026-04-23

**Authors:** Rodrigo-Alejandro Ardiles-Irarrázabal, Miguel Valencia-Contrera, Juan-Carlos Pérez-González, Pablo Pérez-Díaz

**Affiliations:** 1 Departamento de Enfermería, Universidad de Antofagasta, Antofagasta, Chile; 2 Faculty of Nursing, Andrés Bello University, Santiago, Chile; 3 Facultad de Educación, Universidad Nacional de Educación a Distancia (UNED), Madrid, Spain; 4 Faculty of Psychology and Humanities, San Sebastian University, Valdivia, Chile

**Keywords:** emotional intelligence, review, spirituality, students, universities

## Abstract

**Background:**

Research in educational populations has concluded that emotional intelligence positively affects a person’s reality. On the other hand, spirituality has multiple related benefits, such as better quality of life and mental and physical health. However, little evidence has analyzed the relationship between these constructs. The aim of this article was to analyze the existing literature on emotional intelligence and spirituality in undergraduate and postgraduate university students.

**Methods:**

Integrative review, based on the ‘Integra’ strategy with an emphasis on the quality of the results. The WoS, SCOPUS, CINAHL and PubMed databases were consulted; articles in Spanish, English and Portuguese published up to December 2024 were included; letters to the editor, editorials, thesis reports and reviews were excluded.

**Results:**

After analysing the quality of the articles, the final sample consisted of 35 manuscripts published between 2008 and 2024, 33 of which had a quantitative approach. The studies showed significant and positive correlations between emotional intelligence and spirituality. The findings of this study are consistent with those of other authors, highlighting the importance of both constructs in educational systems, given that they allow for the cross-curricular development of multiple dimensions related to students, and may contribute to supporting adaptive behaviors.

**Conclusion:**

Emotional intelligence and spirituality are correlated not only from a theoretical perspective but also empirically, with positive and significant correlations between both constructs, suggesting that they may function as potential protective factors in educational contexts.

## 1 Introduction

The World Health Organization (WHO) defines health as ‘a state of complete physical, mental and social wellbeing, and not merely the absence of disease or infirmity’ (1). This definition recognizes the multidimensional nature of the human being, encompassing mental health and the three domains of the mind: cognitive, affective and behavioral (2), which includes recognition of people’s emotional world. However, this definition lacks, or at least does not explicitly mention, the spiritual dimension. It is therefore crucial to examine how these intangible areas and components of health complement each other.

University students constitute a population increasingly exposed to psychological distress, academic stress, anxiety, depressive symptoms, and burnout. Evidence from a recent review highlights the growing prevalence of mental health problems within this group, positioning university wellbeing as a priority on public health and education policy agendas (3). In this context, identifying protective psychosocial determinants capable of buffering stress and enhancing adaptive functioning becomes strategically essential.

Emotional intelligence (EI) has been consistently associated with stress regulation, resilience, academic adjustment, and psychological wellbeing. Similarly, spirituality, conceptualised as the search for meaning, connection, and transcendence (4); has been linked to mental health, coping ability, and life satisfaction (5). However, despite their conceptual proximity and overlapping protective functions, both constructs have largely been examined independently.

The absence of integrative syntheses examining the relationship between EI and spirituality in the university population limits theoretical integration and hinders the development of comprehensive health promotion strategies in higher education systems.

### 1.1 Emotional intelligence

Emotional intelligence (EI) has established itself as a key concept in psychology, integrating cognition and emotion. There are at least 10 recognized theoretical models in the literature, which share many common elements. In general, these models do not contradict each other but complement each other (6). For example, for Salovey and Mayer, it can be defined as ‘an ability to identify, discriminate, and use one’s own and others’ emotional information to guide thinking, behaviour, and social interactions in an adaptive manner’ (6).

On the other hand, Petrides et al. (7), trait EI (EIt) or trait emotional self-efficacy is defined as a constellation of emotional perceptions assessed through questionnaires and rating scales. Essentially, the construct refers to people’s perceptions of their emotional abilities. It has several factors, which are integrated and considered at the same level of relative importance in contributing to the overall EIt conformation (6).

Related to the above, but from another emotional perspective, academic stress is a systematic process of adaptive physiological and behavioral reactions to academic stimuli and events. University students are prone to develop some level of stress that can trigger emotional, cognitive, psychological and physiological problems, and can even lead to suicide (8). Several studies with higher education populations have shown that EI positively affects a person’s reality (9–11), highlighting the importance of managing emotions appropriately (12). The development of EI is essential in university students, where ways are sought to counteract conditions, improve skills and develop protective factors.

### 1.2 Spirituality

Spirituality is a complex concept (13), an inherent component of human beings that is subjective, intangible, and multidimensional (14). Furthermore, spirituality motivates the search for meaning in life, is universally experienced, and is developed socially and individually throughout existence (15). Spirituality is considered to consist of three main elements: the search for meaning in life, which refers to the reason for being in the world, to a system of goals that fully justifies an individual’s existence; relationships, which include a sense of connection with oneself, with others, with the nature or world, and with a Higher Power, God or Supreme Being; and transcendence, which refers to the ability to change one’s perspective on a given situation and on life in general (13, 15). It also incorporates the capacity for openness, that is, the willingness to encounter, communicate and relate to others (16). Spirituality is therefore a significant component of life that influences emotions, thoughts, and behaviors (17). Furthermore, it is an attempt to give transpersonal, interpersonal and intrapersonal meaning, and has been associated with multiple benefits related to better quality of life, mental and physical health. Spirituality plays an essential role in maintaining mental health and the integral development of university students (16–18). There are few studies on the role of spirituality in university students and its benefits on the health status of this vulnerable population.

Despite the great relevance of emotional intelligence (EI) and spirituality constructs in university students’ health, there are only a limited number of publications that address the apparent complementarity of these constructs. This situation has been described in various reviews of literature, including recent studies covering secondary school students, university students, and health professionals (19, 20). For instance, Vidal’s research only included health-related professions (both studying and working), not considering other disciplines; it is worth remembering that EI and spirituality can contribute to the mental health of future professionals (19). A recent meta-analysis explored the relationship between EI, spirituality, and academic performance (20), but did not consider other aspects related to these constructs and university life, such as health promotion, wellbeing, professional life, etc.

Therefore, the aim of this integrative review was to synthesize and critically analyze the empirical evidence examining the relationship between emotional intelligence and spirituality in undergraduate and postgraduate university students, identifying measurement approaches, disciplinary distribution, and implications for mental health promotion in higher education. Specifically, this review sought to: (a) identify the theoretical models and instruments used to assess EI and spirituality, (b) examine the direction and consistency of associations between both constructs, (c) analyze disciplinary and geographical distribution of studies, (d) explore reported implications for student wellbeing and educational practice.

This integrative synthesis is framed within a mental health promotion perspective in higher education, considering EI and spirituality as potential psychosocial protective determinants.

## 2 Methods

An integrative literature review was conducted, as it distinctly presents a broad approach with diverse sampling, including empirical and/or theoretical literature, thus allowing for a holistic approach to the phenomenon (21).

### 2.1 Design used

This integrative review was conducted and presented in accordance with the global PRISMA 2020 (Preferred Reporting Items for Systematic Reviews and Meta-Analyses) guidelines to ensure methodological transparency. Additionally, the study was structured using the ‘INTEGRA’ methodological strategy (22): [I] Idea or problem to be studied; [N] Question or objective; [T] Search tactics; [E] Execution or use of the search; [G] Degree and quality control of the results; [R] Filtered results; and finally, [A] Analysis and discussion.

[I] Idea or problem to be studied

The topic addressed in this review corresponds to EI and spirituality in undergraduate and postgraduate university students.

[N] Question or objective

The objective of the review was to analyze the emotional intelligence and spirituality of undergraduate and postgraduate university students, regardless of their study of career.

[T] Search strategy

The Web of Science (WoS) database [including KCI-Korean Journal Database, Russian Science Citation Index, and Scielo Citation Index], SCOPUS, Cumulative Index to Nursing and Allied Health Literature (CINAHL), and PubMed were consulted. Data collection was carried out on February 27th 2025, by two authors independently.

In constructing the search equation for selecting descriptors, the Health Sciences Descriptors (DeCS) and Medical Subject Headings (MeSH) thesauri were consulted; pilot tests were conducted with different equations, and the following was ultimately found to be the most effective:((‘Emotional Intelligence’) AND (‘Spirit*’)).

The search strategies and filters used in each database are described below in Table 1.

**Table 1:** Search strategy and filters applied.

Database	Search strategy	Filters applied
WoS	All fields	Languages: English, Spanish, Portuguese Document types: Article Publication years: 2001–2024
SCOPUS	Article title, abstract, keywords	Languages: English, Spanish, Portuguese Document types: Article Publication years: 1999–2024 Keyword: Emotional Intelligence; Spirituality; Students; Student; Nursing Student; Education; Students, Nursing
CINAHL	AB summary	Languages: English, Spanish, Portuguese Publication years: 2002–2024
PubMed	All fields	Languages: English, Spanish, Portuguese Publication years: 2003–2024

Regarding the selection criteria, we included scientific articles with quantitative and qualitative approaches reporting EI and spirituality characteristics in undergraduate or graduate students, in Spanish, English and Portuguese, published until December 2024. Letters to the editor, short communications, abstracts presented at conferences with insufficient information, editorial reviews and meta-analyses, and thesis reports were excluded. Also excluded were those with incomplete information, and inaccessibility of full texts. Studies were retained when emotional intelligence or spirituality were assessed either directly through validated instruments or indirectly through recognised subdimensions theoretically embedded within established EI or spirituality models.

[E] Search execution or use

**Figure 1:**
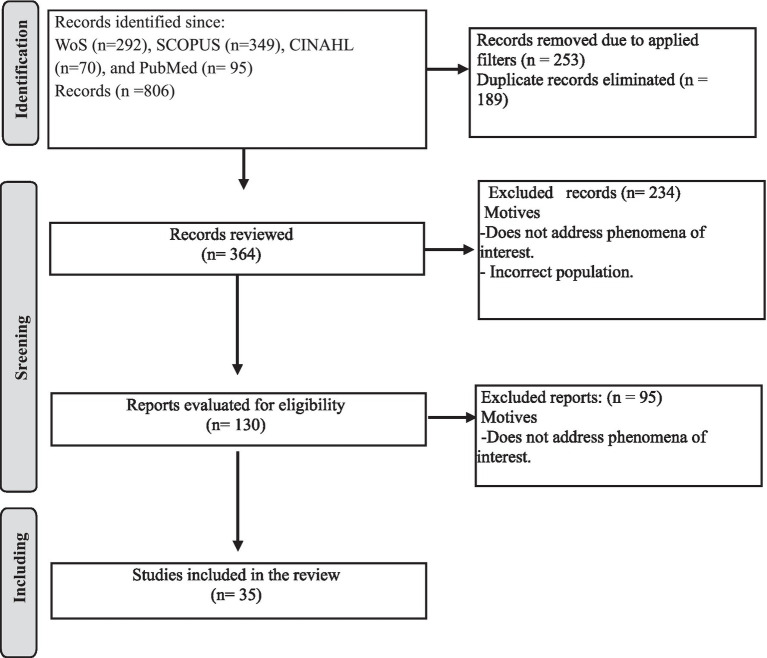
Review flowchart.

After conducting the search, articles that fully met the inclusion criteria were selected. The flow diagram of the review is shown in Figure 1. Two reviewers independently performed the selection of titles/abstracts and the assessment of full-text eligibility. Discrepancies were resolved through discussion and consensus. It was not necessary to consult a third reviewer.

[G] Degree and quality control of results

The EAMH scale has been specifically developed to appraise integrative reviews and has demonstrated methodological coherence in previous applications (22). The instrument evaluates clarity of objectives, methodological coherence, sampling justification, description of data access procedures, and alignment between results and stated aims. Quality assessment was conducted independently by two reviewers following predefined scoring criteria (0–6 points). Discrepancies were resolved through discussion until consensus was reached.

Studies scoring ≥4 points were considered methodologically adequate for inclusion. As shown in Table 2, 10% of manuscripts obtained four points, 37.5% obtained five points (classified as suitable for analysis), and 52.5% obtained six points (classified as ideal for analysis). No studies scored below the predefined adequacy threshold; therefore, all eligible articles were retained in the final synthesis.

**Table 2:** Analysis of the quality of the identified articles.

EAMH scale							
Identified article	Does the article clearly define the objectives?	Does the article clearly define the type of methodology used?	Are the objectives consistent with the methodology used?	Does the article justify the size and type of sample?	Does the article describe how the sample was accessed?	Do the results or conclusions meet the objectives set?	Score 0–6^*^
(23)	1	1	1	0	1	1	5
(24)	1	1	1	1	1	1	6
(25)	1	1	1	1	1	1	6
(26)	1	1	1	1	1	1	6
(27)	1	1	1	1	0	1	5
(28)	1	1	1	1	0	1	5
(29)	1	1	1	1	1	1	6
(30)	1	1	1	1	1	1	6
(31)	1	1	1	1	1	1	6
(32)	1	1	1	1	1	1	6
(33)	1	1	1	1	1	1	6
(34)	1	1	1	1	1	1	6
(35)	1	1	1	1	1	1	6
(36)	1	1	1	0	1	1	5
(37)	1	1	1	1	0	1	5
(38)	1	1	1	0	1	1	5
(39)	1	1	1	1	0	1	5
(40)	1	1	1	0	1	1	5
(41)	1	1	1	0	1	1	5
(42)	1	1	1	0	1	1	5
(43)	1	1	1	1	0	1	5
(44)	1	1	1	1	0	1	5
(45)	1	1	1	0	0	1	4
(4)	1	1	1	1	0	1	5
(46)	1	1	1	1	0	1	5
(47)	1	1	1	1	1	1	6
(48)	1	1	1	0	0	1	4
(49)	1	1	1	0	0	1	4
(50)	1	1	1	1	1	1	6
(51)	1	1	1	1	1	1	6
(52)	1	1	1	1	1	1	6
(5)	1	1	1	1	1	1	6
(53)	1	1	1	1	1	1	6
(54)	1	1	1	1	1	1	6
(55)	1	1	1	1	1	1	6

* 0–3 points: Article not recommended for analysis; 4–5 points: Article suitable for analysis; 6 points: Article ideal for analysis.

Notwithstanding the absence of excluded studies, Table 2 provides a detailed breakdown of scores for each EAMH item, allowing identification of specific methodological limitations across the included studies. In particular, studies that did not achieve the maximum score most frequently lacked adequate justification of sample size and type, as well as a clear description of sample access procedures. These aspects represent relevant methodological weaknesses that should be considered when interpreting the findings, as they may be associated with potential sampling biases and limitations in the transparency of recruitment processes. Accordingly, results derived from these studies should be interpreted with caution (Table 3).

**Table 3:** Characteristics of the articles included.

Country	Author(s) Year	Sample	Language	Instruments	Main results
United States	Smith et al. (2008), (23)	276 undergraduate students	English	Big Five Inventory, Life Orientation Test-Revised. Spirituality and religiosity were assessed using individual items on a five-point scale, Trait Meta-Mood Scale.	Openness to experience, spirituality, and mood attention were the strongest predictors of general willingness to use CAM (Complementary and Alternative Medicine) and were related to willingness to use most individual types of CAM. Advanced age or female gender were associated with a greater willingness to use complementary therapies.
England	Martin and Hafer (2009), (24)	532 business students English Wong Law Emotional Intelligence Survey.	English	Wong Law Emotional Intelligence Survey. Spiritual intelligence was measured using a modified version of the Ashmos and Duchon survey, and performance was the average of the student’s cumulative grade	The under-dimension of IE use of emotion (UOE) negatively impacts performance (GPA). This implies that making intuitive and emotionally triggered decisions about GPA-related schoolwork and topics will not lead to higher GPA performance.
China	Azizollah et al. (2013), (25)	250 undergraduate students	English	Emotional Intelligence Trait Questionnaire - Short Form (TEIque-SF) and the Spiritual Intelligence Self-Report Inventory (SISRI-24)	The best model for predicting educational performance is one that includes different types of intelligence simultaneously. It can be said that emotional and spiritual growth includes personal growth and also goes beyond it.
Canada	Cilliers and Terblanche (2014), (26)	14 fourth-year students	English	Content analysis and positive psychology served as an interpretative lens (qualitative design).	Nursing students experienced identity crises. Recommendations were made for the inclusion of mentoring in the nursing curriculum.
United States	Spofford et al. (2014), (27)	184 psychology students	English	Biosociodemographic questionnaire. Five-Facet Mindfulness Questionnaire (FFMQ), abbreviated version of the Rutgers Alcohol Problem Index. Marlowe-Crowne Social Desirability Scale. Daily Spiritual Experience Scale (DSES).	Religious participants showed higher EI and verbal expression skills, while students who practice meditation showed a higher degree of spirituality. Those who were conscious of not judging reported negative reactions to their own thoughts. The findings do not suggest that mindfulness, social desirability, and religion predict alcohol consumption among African American university students.
United States	Howell and Miller-Graff (2014), (28)	321 undergraduate students	English	Demographic questionnaire. Youth Victimisation Questionnaire - Adult Retrospective - Short Form (JVQR2). Connor-Davidson Resilience Scale (CD-RISC). Depression, Anxiety and Stress Scale - 21 (DASS-21). Life Events Checklist (LEC). Brief Emotional Intelligence Scale (BEIS-10). Daily Spiritual Experience Scale (DSES). Lubben Social Network Scale (LSNS-R)	Greater resilience during emerging adulthood was associated with greater spirituality, higher EI, and support from friends (but not family). The results suggest that the power of protective factors outweighs that of adversity and psychopathology in predicting resilient functioning. By identifying variables that can enhance resilience, this study offers unique insights into how functioning can be improved by both individual and environmental factors.
United States	Beauvais et al. (2014), (29)	124 undergraduate and postgraduate nursing students	English	Mayer-Salovey-Caruso Emotional Intelligence Test (MSCEIT). Spiritual Well-Being Scale (SWBS).	Relationships were identified between emotion management and spiritual wellbeing, and emotion management and existential wellbeing.
Iran	Aghamohammadi and Asgari (2016), (30)	270 medical students	English	NEO-FFI personality trait questionnaires. Emotional intelligence questionnaire by Brad Barry and Jane Graves. Spiritual intelligence scale. Life events scale.	A significant positive relationship was identified between Emotional Intelligence and Spiritual Intelligence with happiness and stress tolerance. Regression analysis also revealed that four-factor emotional intelligence and spiritual intelligence were the best predictors of happiness and stress tolerance.
United States	Gutierrez et al. (2016), (31)	60 master’s level students	English	Demographic questionnaire. Emotional Intelligence Traits Questionnaire – Short Form (TEIque-SF). Perceived Stress Scale. Meditation log.	The results of a randomised controlled trial and growth curve analysis provided a multilevel model in which Jyoti meditation reduced stress and EI moderated the effect. The growth curve supported a moderating effect of EI on student counsellors’ stress levels, indicating that EI has a statistically significant effect on stress and meditation outcomes.
Iran	Heidari Gorji et al. (2017), (32)	324 medical students	English	Bradberry-Greaves Emotional Intelligence Questionnaire. Eysenck Spiritual Intelligence.	The average spiritual intelligence score was higher among women than among men, while the average emotional intelligence score was higher among men. A significant correlation was found between emotional intelligence and spiritual intelligence (r, 0.48; *p* < 0.05). Students’ emotional intelligence can be fostered by strengthening their spiritual intelligence, and correct behaviors in line with social values can be promoted.
Poland	Łowicki and Zajenkowski (2017), (33)	Study 1: 240 university students from various universities in Warsaw, Poland, participated. Study 2: 159 Christian adults, also in Poland, participated.	English	Emotional Intelligence Test (EIT): Measures EI as a skill, assessing perception, understanding, facilitation and management of emotions. Emotional Intelligence Questionnaire as a Trait (TEIQue-SF). Religious Orientation Scale (ROS): Assesses intrinsic religious orientation (religion as an end in itself) and extrinsic religious orientation (religion as a means to other ends). Brief RCOPE: Questionnaire on religious coping strategies, measuring positive and negative coping styles.	IE as a skill is positively associated with the general level of religious beliefs. Both IE as a skill and IE as a trait are negatively correlated with extrinsic religious orientation and negative religious coping. Furthermore, extrinsic religious orientation mediated the relationship between EI as a skill and general religiosity, suggesting that people with high EI may genuinely engage in religion, while those with low EI may have a more instrumental orientation towards religion.
India	Kim (2018), (34)	219 nursing students	English	Spiritual Well-Being Scale (SWB). Connor-Davidson Resilience Scale (CD-RISC). Wong and Law Emotional Intelligence Scale (WLEIS).	Spiritual wellbeing was significantly correlated with resilience (*r* = 0.437, *p* < 0.001) and emotional intelligence (*r* = 0.309, *p* < 0.001). Resilience was also positively and significantly correlated with intelligence (*r* = 0.678, *p* < 0.001). Multiple linear regression showed that religious affiliation (*ß* = 0.544, *p* < 0.001), the subdimension of emotional intelligence ‘use of emotion’ (*ß* = 0.319, *p* < 0.001), and resilience (*ß* = 0.271, *p* < 0.001) were predictors of spiritual wellbeing in nursing students. The spiritual wellbeing of nursing students could be promoted by strengthening resilience, emotional intelligence, and religious affiliation.
India	Yadav and Yadav (2018), (35)	490 undergraduate and postgraduate university students	English	Spiritual Well-Being Scale: It consists of 20 items divided into two subscales: Religious Well-Being (RWB) and Existential Well-Being (EWB). Emotional Intelligence Scale (EIS): Italian version of the scale, with 21 items covering three subscales: Evaluation of Others’ Emotions, Evaluation of Own Emotions, and Regulation and Use of Emotions. Cyberbullying Scale: It consists of 14 items covering two aspects: cyberbullying victimisation and offensive cyberbullying.	A positive relationship was found between Religious Well-Being, EWB Existential Well-Being, AOE Appraisal of Other’s Emotions, ASE Appraisal of Self-Emotions, and RUE Regulation and Use of Emotions. Negative Relationship between Spiritual Well-Being and Cyberbullying: Both spiritual wellbeing and existential wellbeing were negatively related to cyberbullying and cyberbullying victimisation. Mediation by EI: The negative relationships between spiritual and existential wellbeing and cyberbullying and cyberbullying victimisation were significantly mediated by the dimensions of emotional intelligence: Appraisal of One’s Own Emotions, Appraisal of Others’ Emotions, and Regulation and Use of Emotions. Implications: The study suggests that spirituality and emotional intelligence may be effective methods for reducing cyberbullying and victimisation among university students. It is recommended that spirituality be incorporated into programmes and workshops on bullying in schools.
United States	Stewart et al. (2019), (36)	Students: 421 (207 undergraduate and 230 postgraduate) Professionals: 107	English	Toronto Empathy Questionnaire (TEQ): Measures empathy as an emotional process. Big Five Personality Inventory (BFI). Spiritual Beliefs and Involvement Scale (SIBS).	Relationship between Personality and Empathy: The Agreeableness dimension of the Big Five model correlated significantly with empathy. Relationship between Spirituality and Empathy: Only the Spiritual Perspective dimension of the SIBS correlated significantly with empathy. This dimension refers to how spiritual beliefs are applied in daily life and as a coping mechanism. Predictive Impact of Spirituality: Spiritual Perspective added 5% of explained variance in empathy when included in the regression model together with Kindness. Interaction between Personality and Spirituality: The interaction between Spiritual Perspective and Kindness explained an additional 6% of the variance in empathy, suggesting that people with high Kindness may incorporate spirituality into their empathetic responses.
India	Masithah et al. (2019), (37)	300 students	English	Emotional Assessment Scale (AES). Modified Spiritual Intelligence Self-Report Inventory (SISRI).	Emotional and spiritual intelligence significantly influence the intention to quit smoking. The study applied multiple linear regression.
Greece	Fradelos et al. (2019), (38)	206 University students from different departments of the University	English	Hospital Anxiety and Depression Scale (HADS). Trait Emotional Intelligence Questionnaire - Short Form (TEIQue-SF). Functional Treatment of Chronic Illness Spiritual Well-being Scale (FACIT-Sp-12).	There was a positive relationship between emotional intelligence and various factors related to spirituality and psychological health. Relationship between EI and Psychological Stress: A negative correlation was found between anxiety, depression, and EI, suggesting that higher levels of EI are associated with lower levels of psychological stress. Relationship between Spirituality and Psychological Stress: A negative correlation was found between anxiety, depression, and spirituality, suggesting that higher levels of spirituality are associated with lower levels of psychological stress. Factors Associated with Psychological Stress: Factors such as gender, residence, financial status, and educational obligations have a significant impact on the expression of anxiety and depression among students.
Indonesia	Sukartini et al. (2019), (39)	166 clinical nursing students	English	The questionnaire was adopted from Iswanto’s IE. The SI (spiritual intelligence) questionnaire was adopted from Wulandari (2013). The CB (caring behaviour) questionnaire was taken from the Caring, Professional Scale (CPS).	Intelligence quotient (IQ), emotional intelligence (EI), and spiritual intelligence (SI) with prosocial behaviour (CB). The results showed that EI, IQ, and SI are correlated with CB. A higher quotient will produce a higher CB. Future research is expected to analyze other factors related to CC among clinical nursing students. The results showed that CB was correlated with IQ (*p* = 0.019, *r* = 0.211), EI (*p* = 0.048, *r* = 0.178), and SI (*p* = 0.000, *r* = 0.456).
Indonesia	Sari et al. (2019), (40)	336 medical students	English	Emotional instruments include self-awareness, self-assessment, self-confidence, self-control, self-adjustment, self-acceptance, and empathy. An instrument for measuring spirituality that includes awareness, grace, meaning, strength, and truth.	There is a significant relationship between emotional intelligence and spiritual intelligence in student performance index. Students are expected to improve emotional intelligence and spiritual intelligence to solve problems, make decisions, think critically, and think creatively.
Indonesia	Raut and Gupta (2019), (41)	94 medical students	English	Emotional Intelligence Assessment Scale: A self-administered scale of 25 items previously used in India to assess the EI of medical students (no details provided on the instrument used). Reflection Tool: Semi-structured with open-ended questions to trigger students’ thinking process. Peer Feedback Tool: A 20-question Likert scale related to student behaviour.	A significant improvement (*p* < 0.0001) in EI scores was observed at each time point after baseline. Male students from nuclear families who considered themselves spiritual obtained significantly higher median EI scores, and after a mini-workshop there was a significant improvement in EI scores. Facilitating factors: These included personal interest, self-motivation, support from friends and family, openness to learning new things, flexibility for reflection, and choice of peers. Barriers: These included lack of time, lack of mutual trust among students, fear of being judged by peers, non-acceptance of feedback, and limited communication skills.
Portugal	Rodrigues et al. (2019), (42)	345 third-year undergraduate and postgraduate students from various fields of study, including Humanities and Social Sciences, Technological Sciences, Agricultural and Veterinary Sciences, Life and Environmental Sciences, and Nursing	English	Wong and Law Emotional Intelligence Scale (WLEIS). Hodge Intrinsic Spirituality Scale (ISS). Puhakka Creativity Scale. Liñán and Chen PA, PBC and Entrepreneurial Intention Scales (2009).	Relationship between EI and Creativity: EI has a direct positive effect on creativity. Relationship between EI and Personal Attitudes towards Entrepreneurship (PA): EI has a direct positive effect on personal attitudes towards entrepreneurship. Relationship between EI and Perceived Behavioural Control (PBC): EI has a direct positive effect on perceived behavioural control. Relationship between PA and PBC with Entrepreneurial Intention: Personal attitudes towards entrepreneurship and perceived behavioural control have a direct positive effect on entrepreneurial intention. Mediating Effect of PA and PBC: Personal attitudes towards entrepreneurship and perceived behavioural control mediate the effect of emotional intelligence on entrepreneurial intention. Influence of Spirituality: Spirituality did not show a significant influence on creativity or entrepreneurial intention, and only had a weak impact on personal attitudes towards entrepreneurship and perceived behavioural control; there was no statistically significant correlation between EI and spirituality.
Australia	Pant and Srivastava (2019), (43)	300 graduate students	English	Integrated Spiritual Intelligence Scale (ISIS). Mithila Mental Health Status Inventory (MMHSI).	Relationship between SI and MH: There is a significant relationship between spiritual intelligence and mental health in both arts and science students. Gender Differences: No significant differences were found between men and women in terms of spiritual intelligence and mental health. Differences by Discipline: No significant differences were found between arts and science students in terms of spiritual intelligence and mental health.
India	Ganguly and Perera (2019), (44)	274 university students registered with the Division of Disability Services at a regional university	English	Resilience was measured using the CD-RISC. CO was measured using the *CO subscale of the Career Futures Inventory. AS was measured using the Major Academic Satisfaction Scale. WHO-5 wellbeing; WHO-5. *CO: refers to favourable expectations of success for career-related development.	Resilience Profiles: Three distinct resilience profiles were identified: ‘vulnerable,’ ‘spiritually dominant,’ and ‘resiliently committed.’ Gender and Resilience: Women were nearly four times more likely to be in the ‘spiritually dominant’ profile than in the ‘vulnerable’ profile. Profile Results: Career optimism, academic satisfaction, and psychological wellbeing were higher in the ‘resilient committed’ profile than in the other profiles. ‘Spiritually dominant’ and ‘vulnerable’ individuals had similar levels of career optimism, satisfaction, and wellbeing.
Venezuela	Vakili and Zaré (2019), (45)	32 students of education sciences and psychology	English	Schutte’s 4-scale emotional intelligence questionnaire. 4-scale spirituality questionnaire (SQ).	The findings showed that spiritual intelligence does not affect emotional intelligence. In conclusion, the importance of spiritual beliefs in life has no effect on students’ emotional intelligence.
Chile	Ardiles-Irarrázabal et al. (2020), (4)	184 first- to fifth-year nursing students	Spanish	Spirituality Questionnaire SQ, Chilean version. TMMS-24	The correlation reported a moderately low association between the dimensions of spirituality and two of the three dimensions of emotional intelligence, namely emotional understanding and regulation. It is concluded that there is a relationship between the constructs. Furthermore, promoting training in at least one of the variables contributes to the human development of nursing students, providing space to offer more comprehensive and multidimensional care to our users.
Poland	Samul (2020), (46)	190 undergraduate students from the Faculty of Management Engineering	English	Spiritual Intelligence Inventory (SISRI-24). Emotional Intelligence Scale (WLEIS). Self-Leadership Questionnaire (SL).	Relationship between EI and SL: Emotional intelligence has a positive relationship with self-leadership. Relationship between SI and SL: Spiritual intelligence also has a positive relationship with self-leadership. Relationship between EI and SI: There is a positive relationship (*r* = 0.508) between emotional intelligence and spiritual intelligence, although emotional intelligence is not a significant predictor of spiritual intelligence. Levels of EI and SI: Students’ levels of emotional and spiritual intelligence were assessed as average, suggesting a need to strengthen these skills in leadership education programmes.
Malaysia	Anwar et al. (2020), (47)	250 university students from various higher education institutions	English	Spiritual Intelligence Questionnaire from an Islamic perspective. Wong and Law Emotional Intelligence Scale (WLEIS).	Relationship between SI and EI: There is a significant relationship between spiritual intelligence from an Islamic perspective and emotional intelligence. Dimensions of SI and EI: The dimensions of transcendental awareness, meaning of life, patience, and forgiveness have significant relationships with emotional intelligence. Impact of SI on EI: Spiritual intelligence from an Islamic perspective positively influences students’ emotional awareness and control.
Indonesia	Turi et al. (2020), (48)	113 students	English	Spiritual Well-Being Scale. Emotional Intelligence Questionnaire	The findings provide positive and significant correlations between types of intelligence and academic performance. The correlations show the relationship of significance between the elements of intelligence and show that each independent variable has a significant relationship with the dependent variable, academic performance. These correlations indicate that better integration improves academic practice experiences, routine, and culture.
United States	Perkins and Aquino-Russell (2020), (49)	Doctoral students in nursing who practised Transcendental Meditation (TM) for 4 months. The exact number of participants is not specified.	English	Phenomenological design to explore the lived experiences of doctoral students in nursing who practised Transcendental Meditation (TM) for 4 months. Instruments used. Transcendental Meditation (TM): A meditation technique used by participants. Self-report: Participants described their experiences and perceived changes in their emotional and spiritual intelligence, resilience to stress and anxiety, and development of mindfulness.	Consciousness Development: The practice of TM facilitated the development of expanded consciousness and authentic presence in participants. Emotional and Spiritual Intelligence: Participants reported improvements in their emotional and spiritual intelligence, as well as greater resilience to stress and anxiety. Values and Virtues: Practising TM helped participants develop and live values and virtues such as trust, balance, forgiveness, love, and creativity.
United States	Sturgill et al. (2021), (50)	99 first-year university students at a liberal arts institution	English	Test Well-being Inventory. The Patient Health Questionnaire (PHQ-9) 7-item scale for Generalised Anxiety Disorder (GAD-7) Emotional Quotient There are several measures of EI Ajivar: A life coach powered by EI and AI (mindfulness training)	Students in the intervention group who used the Ajivar app showed a significant decrease in anxiety and depression levels, as measured by the GAD-7 and PHQ-9. The mean GAD-7 score decreased from 11.47 at the start of the study to 6.27 at the end (P < 0.001). The mean PHQ-9 score decreased from 10.69 at the start of the study to 6.69 at the end (*p* < 0.001). EQ scores increased from 62.87 at the start of the study to 71.17 at the end (p < 0.001). Significant improvements in the social awareness and spirituality subcategories in the intervention group. The Ajivar app proved effective for delivering emotional intelligence training and mindfulness techniques, especially during the COVID-19 pandemic.
Pakistan	Jafari et al. (2021), (51)	136 nursing students	English	The Bradberry and Greaves El scale. Palutzian and Ellison’s Spiritual Well-Being Scale (SWB) to assess the total score for El and SWB.	Most students reported moderate SWB and high emotional intelligence. The factors that influenced their SWB level were the academic semester and age (p < 0.05). Although the students’ El and SWB levels were at a desirable level in this study, due to the nature of nursing and the interaction between nurses and patients, it is essential to provide an appropriate learning environment for the development of EI.
United States	Zhang et al. (2023), (52)	1,811 nursing practitioners	English	Demographic questionnaire. Competency inventory for the Registered Nurse Questionnaire (CIRN), the Chinese version of the Spiritual Care Scale Questionnaire (C-SCGS) and the Chinese version of Wong and Law’s Emotional Intelligence Scale Questionnaire (WLEIS-C).	The core nursing competencies (NCC) of university nursing interns were related to whether they worked as student leaders, had a better level of self-assessment, were university nursing interns with good interpersonal relationships, and intended to practice nursing in the future. The NCC, IE, and SCG scores were (156.43 ± 23.14), (61.55 ± 9.10), and (167.64 ± 20.52), respectively. Positive correlations were observed between SCG (*r* = 0.402), IE (*r* = 0.506), and NCC. The partial mediating effect of EI between SCG and NCC was 0.127, representing 36.29% of the total results.
Israel, India	Walter et al. (2024), (5)	554 university students	English	Demographic survey. Emotional Self-Efficacy Scale (ESES), a self-report measure. Spiritual Self-Assessment Inventory (SISRI-24). 4-item Patient Health Questionnaire (PHQ-4) Life Satisfaction Scale	In Israel, emotional intelligence was positively correlated with life satisfaction, while in India, only spiritual intelligence was positively correlated with life satisfaction. Women scored higher on all measures in both countries. Emotional intelligence and spiritual intelligence predicted life satisfaction, but emotional intelligence had a greater impact in Israel, while spiritual intelligence had a greater impact in India.
Indonesia	Sojer et al. (2024), (53)	277	English	Self-Report Emotional Aptitude Scale (SEAS), developed by Freudenthaler and Neubauer to assess facets of EI as a trait. Connor-Davidson Resilience Scale (CD-RISC) (includes spiritual influence).	With respect to trait EI, women scored significantly higher on the total interpersonal emotional skills score and on the subscale ‘Perception of others’ emotions’ than men. Men showed significantly higher total scores on intrapersonal emotion-related skills than women, and on the subscales ‘Regulation of own emotions’ and ‘Control over expression of own emotions’. With respect to resilience, female students had significantly higher scores on the CD-RISC subscales ‘Personal competence and tenacity’, ‘Control’ and ‘Spiritual influence’. The sum score of the intrapersonal trait EI (SEAS) showed a significant positive correlation with the total scores of the CD-RISC (rs = 0.445, *p* < 0.001). There were also positive correlations between the interpersonal trait EI sum score and the CD-RISC total score (rs = 0.438, *p* < 0.001).
	Raheja et al. (2024), (54)	240 undergraduate	English	Oxford Happiness Questionnaire. D. King’s SISRI-24 Spiritual Intelligence Measurement Scale. Academic Achievement Assessment Questionnaire (designed by the authors).	Spiritual intelligence and students’ happiness are positively and significantly related to each other both in general and specifically for males and females, at a significance level of 1%. This association was more prominent for female students.
eru	Remaycuna-Vasquez et al. (2024), (55)	600 undergraduates from private universities	Spanish	Spiritual Intelligence Scale SISRI-24 Alarcón Happiness Questionnaire	34.5% of the students need to improve their spiritual intelligence, while 35.5% have low levels of happiness. There is a significant relationship between spiritual intelligence and happiness (*p* = 0.000). There are no significant differences between spiritual intelligence and happiness in terms of gender, but there are significant differences in terms of age; students older than 25 years develop a better capacity for spiritual intelligence.

## 3 Results

**Figure 2:**
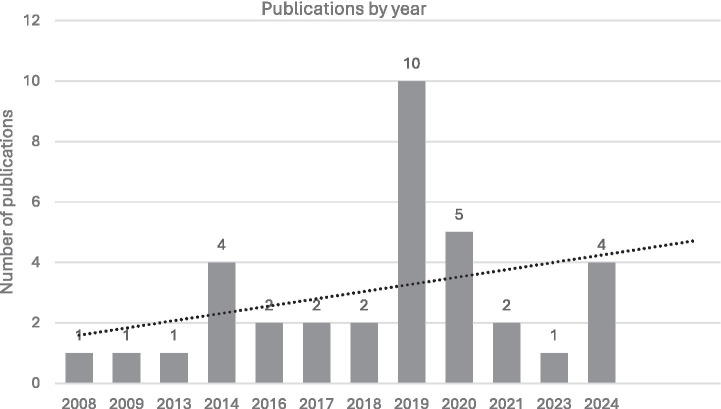
Number of articles identified by year of publication.

To comply with the penultimate stage of review, ‘filtered results’ (22), present the studies that form part of the final sample, i.e., 35 articles (4, 5, 23–55). Regarding time range, the articles spanned from 2008 to 2024 (Figure 2); two of them had a qualitative approach (26, 49); and only two were in a language other than English (4, 55).

Most studies were conducted in the United States (23, 27–29, 31, 36, 49, 50, 52), followed by India (34, 35, 37, 44, 54) and Indonesia (39–41, 48, 53), accounting for 54.28% of the studies included in the review. Poland (33, 46), and Iran (30, 32) contributed two studies. England (24), China (25), Canada (26), Greece (38), Australia (43), Venezuela (45), Chile (4), Malaysia (47), Pakistan (51), Israel-India (5), Peru (55), contributed only one study each.

Research examining the relationship between EI and spirituality shows a general tendency towards positive associations; however, given the heterogeneity in study designs, instruments, and analytical approaches, findings were synthesised narratively rather than through vote counting. Studies using trait and mixed EI models (e.g., TEIQue, WLEIS) tended to report more consistent positive associations with spirituality-related constructs, particularly those linked to perceived emotional self-efficacy and wellbeing. In contrast, studies based on ability models reported positive but comparatively more moderate associations. From the perspective of spirituality measurement, studies using constructs such as spiritual wellbeing and spiritual intelligence generally identified positive relationships with EI, particularly in relation to psychological wellbeing and resilience, whereas studies using more context-dependent constructs (e.g., religiosity or meditation) reported more variable findings. Additionally, some studies identified more complex relationships, including moderation and mediation effects, as well as associations with outcomes such as stress, cyberbullying, and academic performance. Qualitative studies also supported interpretative linkages between both constructs.

Importantly, spirituality was not treated as a homogeneous construct across the included studies. Instead, the instruments used operationalised different dimensions, including spirituality as a state (e.g., spiritual wellbeing), as a capacity or trait (e.g., spiritual intelligence), and as context-dependent expressions or practices (e.g., religiosity, meditation, or daily spiritual experiences). When considering these distinctions, studies assessing spirituality as a state tended to report associations with emotional outcomes such as wellbeing, stress, and resilience. In contrast, studies conceptualising spirituality as a capacity showed closer alignment with EI dimensions related to emotional regulation and self-efficacy. Finally, studies focusing on behavioural or experiential aspects of spirituality reported more variable associations, depending on contextual and cultural factors.

In relation to assessment instruments in research, eight different instruments were recorded for EI, notably those related to Wong and Law (WLEIS), with six studies (24, 34, 42, 46, 47, 52), followed by those related to TraitEI, with five articles (25, 31, 33, 38, 53), and some articles were included because they were part of the facets of EI theory (wellbeing, happiness, empathy, etc.) with 5 studies (36, 43, 44, 54, 55), in addition to those related to Salovey and Mayer’s theories with 3 studies (4, 23, 29). Another EI instrument used in three studies was the ‘Brad Barry and Jane Graves Emotional Intelligence Questionnaire’ (30, 32, 51). Others had non-specific information (40, 41, 48), and other scales that were only used once (5, 28, 35, 37, 39, 45, 50). When differentiating the findings according to the theoretical EI model used, there is a predominance of research using the EI-Trait model (*n* = 5), assessed mainly through the Trait Emotional Intelligence Questionnaire (TEIQue), and mixed models such as Wong and Law (WLEIS) (*n* = 6). In these studies, correlations with spirituality tended to be consistently positive and significant. In contrast, research based strictly on Salovey and Mayer’s ability model (*n* = 3) reported positive associations, albeit with more moderate effect sizes compared to self-report models. This distinction suggests that spirituality is more strongly associated with perceived emotional self-efficacy (trait) than with cognitive-emotional executive capacities (ability), without implying conceptual overlap between these constructs, which remain theoretically distinct. Likewise, two publications did not use instruments due to their qualitative design (26, 49). Finally, it should be noted that one research did not report on the EI instrument used, but was included anyway because its approach, findings and the constructs used were taken into account (27).

On the other hand, the instruments used in spirituality included the Spiritual Intelligence Inventory (SISRI-24) in seven studies (5, 25, 30, 37, 46, 54, 55); another scale used was the Spiritual Well-Being Scale (SWBS) in five articles (29, 34, 35, 48, 51); likewise, the Spirituality Questionnaire (SQ) was used in 2 articles (4, 45). Two others used the Daily Spiritual Experience Scale (DSES) (27, 28). In addition, two used the Connor-Davidson Resilience Scale (CD-RISC) (including spiritual influence) (44, 53). There are 13 articles with a single spirituality instrument (23, 24, 32, 33, 36, 38–40, 42, 43, 47, 50, 52), and finally, there are four articles that do not record instruments, two due to their qualitative design (26, 49). Seven studies included postgraduate students, and 31 included undergraduate students. Some studies involved mixed samples comprising both undergraduate and postgraduate participants; therefore, these categories are not mutually exclusive. The seven (29, 31, 35, 36, 42, 43, 49); and 31 with undergraduate students (4, 5, 23–30, 32–42, 44, 46–48, 50–55).

In relation to the disciplines of the students who related both variables, the 12 studies in the area of health (4, 26, 29, 30, 32, 34, 36, 39, 42, 49, 51, 52) stand out (34.3% of the total). Publications on undergraduate and postgraduate nursing students stand out, with 10 studies from different locations (4, 26, 29, 34, 36, 39, 42, 49, 51, 52). In addition, four studies were reported from medicine students (30, 32, 40, 41). There were also two articles that appraised psychology students (27, 45), and one study each, that assessed business (24), education (45), and engineering (46) populations.

The current research can be framed to the following topics: 32 articles related to lifestyle correlates and psychospiritual health (4, 5, 23, 26–39, 41–47, 49–55), 6 articles focused on risk factors (e.g., stress, depression.) (26, 30, 31, 38, 49, 50); and 4 related to academic performance (24, 25, 40, 48).

## 4 Discussion

### 4.1 Interpretative synthesis of findings

This integrative review suggests that emotional intelligence (EI) and constructs linked to spirituality have a predominantly positive association in the university population. After analysing the body of evidence from 2008 to 2024, most studies report statistically significant correlations, while a minority report no associations. Beyond linear correlation patterns, several studies explored complex relational dynamics, such as moderation and mediation effects between EI dimensions and spiritual variables.

These findings suggest that the link between the two domains operates through multiple psychosocial pathways critical to student wellbeing, reinforcing the hypothesis that EI and spirituality may interact in a complementary manner in the development of adaptive resources.

It is important, however, to consider that the convergence observed may partially reflect shared variance derived from self-report methodologies and overlapping subdimensions (e.g., self-awareness, emotional regulation, meaning-making). Therefore, some of the positive associations reported might be inflated by conceptual proximity rather than representing fully independent psychosocial mechanisms.

The concentration of studies within health disciplines, particularly nursing, likely reflects the formal integration of holistic care and spiritual competencies within these curricula. However, this disciplinary predominance may limit the generalisability of findings to other academic contexts where spirituality is conceptualised differently.

### 4.2 Conceptual and measurement heterogeneity: implications for synthesis

Despite convergence in the direction of associations, direct comparability between studies is compromised by substantial heterogeneity. First, EI was operationalised using divergent theoretical models, including trait-based approaches (self-report measures) and skill-based approaches (performance). This distinction is ontologically relevant: while trait EI captures perceived emotional self-efficacy, skill EI assesses effective cognitive-emotional capacities, preventing estimates of effects from being interchangeable.

Second, spirituality was addressed using instruments that capture heterogeneous constructs, such as spiritual wellbeing, spiritual intelligence, daily transcendental experiences, or the search for meaning, limiting conceptual equivalence. Finally, the communication of results showed inconsistencies in the statistical indicators provided (correlations, regression coefficients, mediation paths) and in the control of confounding variables. These sources of variability justify the methodological decision to perform an integrative narrative synthesis rather than a quantitative meta-analysis; an estimate of the combined effect under these conditions would be spurious as it would amalgamate non-equivalent measurement models.

Additionally, the reporting inconsistency of statistical parameters (e.g., absence of effect sizes, confidence intervals, or standardised coefficients in several studies) precluded the computation of pooled estimates or heterogeneity statistics (I^2^ or Q). Therefore, quantitative aggregation would have risked producing misleading summary effects. Moreover, the predominance of cross-sectional designs prevents temporal inference and restricts conclusions regarding directional influence between constructs. Whether spirituality enhances emotional intelligence, emotional intelligence fosters spiritual development, or both are shaped by third variables (e.g., personality traits, cultural context) remains unresolved.

### 4.3 Analysis and critical discussion: mental health and academic performance

In line with the stages of analysis and discussion proposed by Valencia-Contrera et al., this research reinforces the robustness of the relationship between EI and spirituality across various disciplines, years, and educational levels (22). Certain studies have linked these constructs to academic performance (24, 25, 40, 48), findings that converge with the recent meta-analysis (20); which highlights that strengthening emotional and spiritual intelligence promotes academic success.

Likewise, most of the studies included (*n* = 32) linked these variables to lifestyle and psychospiritual health (23, 26–31) among others included in the final sample. These results are consistent with previous research in similar populations (9–12, 16–18), highlighting the benefits of these constructs for personal and professional development.

From a health promotion perspective, EI and spirituality emerge as protective factors against stress, anxiety and tension (26, 30, 31, 38, 49, 50). From a broader theoretical perspective, the integration of EI and spirituality may be interpreted within contemporary models of positive psychology and eudaimonic wellbeing, where emotional regulation and meaning-oriented processes operate synergistically. However, future research should clearly distinguish between hedonic, eudaimonic, and transcendental dimensions in order to avoid conceptual blending and artificial inflation of associations.

The predominance of health-related samples reinforces the relevance of psycho-spiritual competencies in professions exposed to emotional stress. However, this disciplinary approach also suggests the need for broader exploration in non-health-related faculties. This coincides with Vidal Barrantes in that these attributes protect professionals from the emotional burden inherent in the suffering of others (19). The relevance observed in nursing (4, 26, 29, 34, 36, 39, 49, 51, 52) confirm that spiritual care is an intrinsic component of holistic care, validating the need to optimise psycho-spiritual competencies to improve the quality of care and student wellbeing (4, 56).

### 4.4 Implications for practice and higher education

The intersection between EI and spirituality offers a strategic framework for mental health policies in higher education. Universities represent ideal environments for the implementation of large-scale programmes for emotional regulation, adaptive coping, and the search for meaning. The available evidence suggests the relevance of integrating emotional skills training with reflective components oriented towards life purpose within student wellbeing initiatives.

Although the nature of the evidence does not allow for the establishment of causality, the findings point to plausible directions for health promotion. Institutions could pilot programmes that combine the development of emotional competence with structured practices on values and transcendence, assessing their feasibility and acceptability before systemic implementation. This position is reinforced by studies demonstrating the positive impact of EI in educational settings (9–11) and its essential role in maintaining overall health (16–18).

### 4.5 Strengths, limitations and future agenda

A key strength of this study is the comprehensive synthesis of evidence from high-impact databases (WoS, Scopus, CINAHL and PubMed), constituting, to our knowledge, one of the first integrative reviews to systematically analyze the link between these constructs in undergraduate and postgraduate university students. However, limitations are acknowledged: the exclusion of grey literature and language restrictions may have introduced selection and publication biases.

In addition, although the selection of databases was intentionally focused on high-impact and discipline-relevant sources to ensure methodological rigour and reproducibility, the non-inclusion of other multidisciplinary and complementary databases such as JSTOR, ProQuest, PsycINFO, and Google Scholar may have limited the identification of additional studies from adjacent fields (e.g., social sciences, psychology, and education). Future reviews could benefit from incorporating these sources to broaden interdisciplinary coverage and enhance comprehensiveness.

Furthermore, the search strategy employed, based on the combination of “Emotional Intelligence” AND “Spirit,” may have introduced an epistemological bias by privileging literature grounded in holistic and spiritual care frameworks, which are more prevalent in health-related disciplines. This may have limited the retrieval of studies from other fields—such as psychology, engineering, or business—where related constructs are often operationalised using alternative terms (e.g., religiosity, meaning in life, existential wellbeing). Consequently, despite the intention to include students regardless of their field of study, the resulting sample shows a greater representation of health disciplines, which should be considered when interpreting the findings. Future reviews should incorporate broader and more inclusive search terms to ensure a more balanced interdisciplinary representation.

Furthermore, the predominance of cross-sectional designs limits temporal inference. Additionally, the frequent reliance on convenience samples drawn from single institutions limits external validity. Cultural and religious contexts may substantially shape the operationalisation and expression of spirituality, suggesting that cross-cultural comparative designs are necessary to strengthen generalisability.

Finally, it should be noted that the search strategy was conducted in February 2025, and although the manuscript was submitted in February 2026, a formal update of the search was not performed. This time lag may have led to the omission of more recent studies published during late 2025 and early 2026. However, given the consistency of the findings observed across the included literature and the integrative nature of the review, it is unlikely that newly published studies would substantially alter the main patterns identified. Nevertheless, future updates of this review are recommended to incorporate emerging evidence and ensure the continued relevance of the findings.

The absence of registration in PROSPERO was due to the integrative and heterogeneous design of the study; however, transparency was ensured through the *a priori* definition of protocols, inclusion criteria, and search strategies.

In line with this limitation, future research could also benefit from conducting comparative analyses between health and non-health disciplines to explore potential disciplinary differences in the relationship between emotional intelligence and spirituality, thereby enhancing the interpretative depth and novelty of the field.

For future research, the following are prioritised: (1) rigorous conceptual alignment between EI and spirituality models; (2) standardisation of statistical indicators to facilitate higher-level synthesis; (3) the adoption of longitudinal designs; and (4) the development of intervention studies that evaluate specific mechanisms (regulation, meaning, social connection) and their impact on academic and mental health outcomes, using both robust quantitative approaches and qualitative approaches that capture the depth of subjective experience.

## 5 Conclusion

This study provided answers to the proposed objectives, analysing the emotional intelligence and spirituality of undergraduate and postgraduate university students. It concludes by highlighting the similarity of both constructs, not only at a theoretical and qualitative level (from self-awareness, interpersonal relationships, etc.), but also showing that most studies reported positive associations, although heterogeneity in measurement approaches limits direct comparability. Furthermore, strong associations were observed between related common criteria, mediating predictors, and moderating correlates. Therefore, there is not only a theoretical link, but also an empirical relationship.

The practical implications are particularly evident in health disciplines, where psycho-spiritual competencies appear closely integrated with professional formation. EI and spirituality may act as potential protective factors against the negative emotional burden experienced by these students.

Finally, and given the evidence of the present and other related research, universities and academics may play a relevant role in promoting spaces for the development of EI and spirituality, which could contribute to reducing negative psycho-spiritual outcomes and enhancing positive developmental processes among university students.
